# Minimally Invasive Treatment of Mirizzi Syndrome, a Rare Cause of Cholestasis in Childhood

**DOI:** 10.1155/2016/8940570

**Published:** 2016-10-24

**Authors:** Ahmet Ali Tuncer, Sezgin Yilmaz, Mustafa Yavuz, Salih Çetinkurşun

**Affiliations:** ^1^Department of Pediatric Surgery, Afyon Kocatepe University Hospital, Afyonkarahisar, Turkey; ^2^Department of General Surgery, Afyon Kocatepe University Hospital, Afyonkarahisar, Turkey

## Abstract

Mirizzi syndrome is the compressive blockage of the cystic or choledochal duct caused by a biliary stone occupying the cystic canal or Hartmann's pouch. This occurrence is rare and, in English literature, three cases defined in children have been observed. In order to draw attention to this rare occurrence, we preferred a 14-year-old male patient with Mirizzi syndrome. In this case, ERCP was performed preoperatively and the diagnosis was carried out with the help of clear visualisation and identification of the tissue structures as well as the stent placed in bile duct; so we protected the patient from the possible iatrogenic injury occurring during surgery.

## 1. Introduction

Mirizzi syndrome is the compressive blockage of the cystic or choledochal duct caused by a biliary stone occupying the cystic canal or Hartmann's pouch. It was first described in 1948 by Mirizzi [1], clinically characterized by intermittent or persistent obstructive jaundice. It is more common in patients with cholelithiasis for a long time and women [2]. There are three cases defined in children in English literature [3–5]. Factors that make this patient population special is the difficulty in preoperative diagnosis and treatment, with an additional high risk of iatrogenic damage to the biliary system. In order to draw attention to this rare occurrence, we preferred a 14-year-old male patient with Mirizzi syndrome.

## 2. Case Presentation

**Figure 1:**
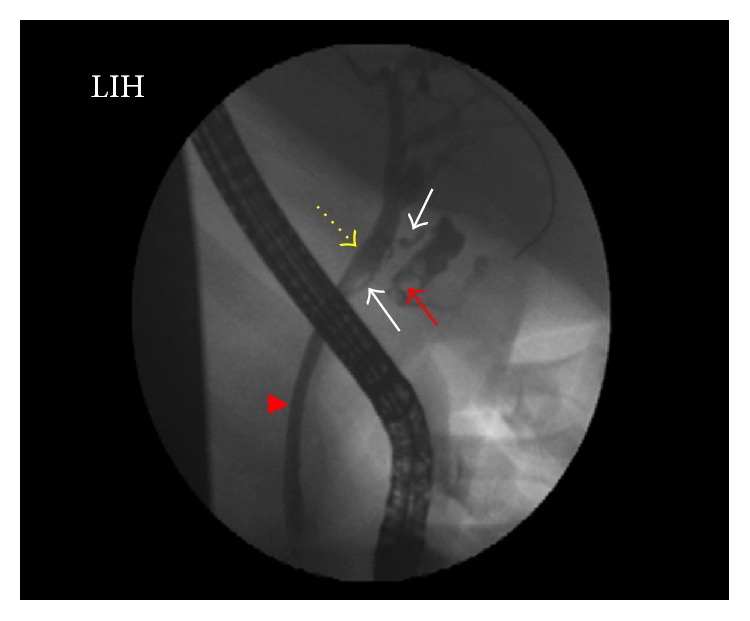
ERCP shows Mirizzi syndrome (arrow head: ductus choledochus, yellow dotted arrow: dilated common hepatic duct, white arrows: cystic duct, and red arrow: gallstones in Hartmann's pouch).

**Figure 2:**
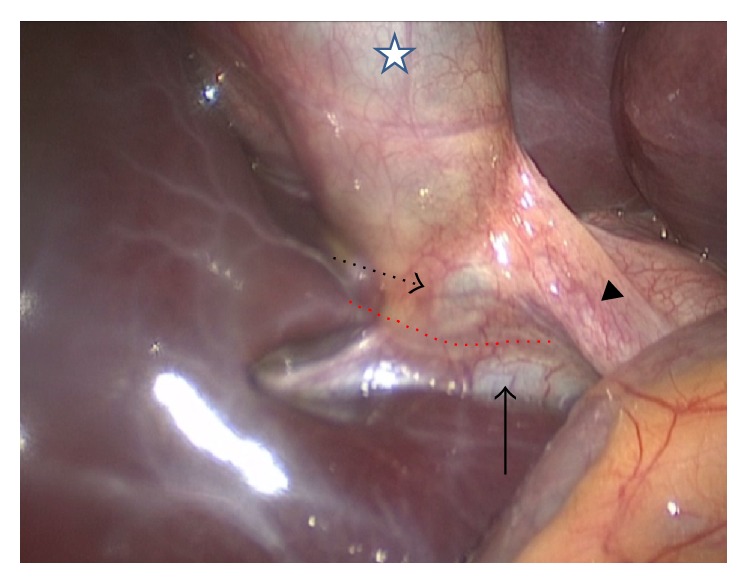
Laparoscopic overview of the Mirizzi syndrome (star: gallbladder, arrow head: cystic duct, dotted arrow: Hartmann's pouch, arrow: common hepatic duct with stent, and dotted red line: the incision plan between Hartmann's pouch of gallbladder and common hepatic duct).

A fourteen-year-old male patient applied to our clinic with a 1-week history of right upper quadrant pain and icteric sclera. Physical examination revealed the tenderness on Murphy's point and jaundice extending down to the body. Laboratory examinations were consistent with obstructive jaundice (aspartate aminotransferase: 141 U/L, alanine aminotransferase: 230 U/L, gamma-glutamyl transpeptidase: 219, total bilirubin: 7.81 mg/dL, conjugated bilirubin: 7.4 mg/dL, white blood cell: 12.1 × 10^3^/*μ*L, and neutrophil: 8.9 × 10^3^/*μ*L). After ultrasonography was performed on the patient that revealed cholelithiasis and cholestasis, endoscopic retrograde cholangiopancreatography (ERCP) was planned. During ERCP, it seemed that the cystic duct is quite weak and inside Hartmann's pouch compressed the choledoc filled with stones. Sphincterotomy was carried out. After the choledochal canals were cleaned, a choledochal stent was replaced (Figure 1). Laparoscopic cholecystectomy was carried out a day later. During laparoscopy, choledochal compression caused by Hartmann pouch was clearly seen (Figure 2). The patient was discharged after being observed to have normal postoperative bilirubin values. Choledochal stent was taken by endoscopy on the first postoperative month. In Mirizzi syndrome since the gallbladder compresses the choledochus and eventually causes obstructive jaundice, it should be removed. Since the patient was admitted with cholecystitis and obstructive jaundice, a preoperative ERCP should be planned in order to normalize the jaundice and clarify the etiology as in our patient. An immediate laparoscopic cholecystectomy should be planned just after the ERCP. Cholecystectomy was carried out under general anesthesia but endoscopies were performed by means of sedation. The patient was followed up for three months without any complications.

## 3. Discussion

Mirizzi syndrome's incidence is 0.06%–2.7% in adult patients who underwent cholecystectomy, seen in a rare entity [6]. However, it is extremely rare in children and it requires sharing information with general surgeons and pediatric surgeons in these patients.

Imaging methods such as ultrasound, computed tomography, endoscopic retrograde cholangiopancreatography (ERCP), and magnetic resonance cholangiopancreatography (MRCP) provide benefits in establishing preoperative diagnosis in patients with clinical findings of obstructive jaundice [3, 5]. Even so, preoperative diagnosis is in 50% of cases. In our case, there were symptoms of obstructive jaundice and abdominal pain. Because cholestasis and cholelithiasis-induced bile duct obstruction are suspected in ultrasonography, ERCP was carried out. Mirizzi syndrome diagnosis seemed clear in ERCP. The sample is going to be known as preoperative guidance to the surgeon because classification of Mirizzi syndrome affects the surgical approach. Csendes et al. had classified Mirizzi syndrome as type 5 [2, 7]. Type 1 indicates obstruction of the hepatic duct by an impacted biliary stone in Hartmann's pouch or in the cystic duct; type 2 indicates that biliary stone erodes the bile duct causing a cholecystobiliary fistula containing less than one-third of the circumference of the bile duct; type 3 means cholecystobiliary fistula involves two-thirds of the circumference of the bile duct; type 4 means cholecystobiliary fistula with whole destruction of the bile duct wall and dissection plane is unrecognizable; type 5 indicates presence of cholecystoenteric fistula. Our case was consistent with type 1 [2, 7].

The treatment of Mirizzi syndrome is surgical. The main factor that determines the surgical method is the occurrence of the fistula. Open or laparoscopic cholecystectomy is preferred in type 1 Mirizzi syndrome. In case of the fistula occurrence, bile duct intervention should be done by open surgery method. If the defect is small in type 2, common bile duct repair and total or subtotal cholecystectomy can be done through T tube. Choledochoduodenostomy or Roux-en-Y hepaticojejunostomy may be preferred in 3-4 types [8].

The risk of bile duct injury during laparoscopic cholecystectomy is reported to be from 0.3% to 1.0% [9]. However, in operations without confirmed preoperative diagnosis such as Mirizzi syndrome, this risk goes up to 17% [4]. Intraoperative cholangiography and ultrasound can be used to confirm the diagnosis or to determine the presence and extent of biliary fistula.

In this case, ERCP was performed preoperatively and the diagnosis was carried out with the help of clear visualisation and identification of the tissue structures as well as the stent placed in bile duct; so we protected the patient from the possible iatrogenic injury occurring during surgery. Also cholecystectomy was performed after ERCP in the first days that kept the patient from possible cholecystitis due to opaque material. Mirizzi syndrome should be kept in mind as one of the differentials in patients whose diagnoses are hindered due to potential difficulties in treatment planning, and the possibility of serious iatrogenic biliary damage inflicted during surgery should be taken into consideration.
